# Comprehensive Analysis of *Eleutherococcus senticosus* (Rupr. & Maxim.) Maxim. Leaves Based on UPLC-MS/MS: Separation and Rapid Qualitative and Quantitative Analysis

**DOI:** 10.3389/fphar.2022.865586

**Published:** 2022-05-17

**Authors:** Jianping Hu, Dan Wu, Yanping Sun, Hongquan Zhao, Yangyang Wang, Wensen Zhang, Fazhi Su, Bingyou Yang, Qiuhong Wang, Haixue Kuang

**Affiliations:** ^1^ Key Laboratory of Basic and Application Research of Beiyao, Ministry of Education, Heilongjiang Touyan Innovation Team Program, Heilongjiang University of Chinese Medicine, Harbin, China; ^2^ Medical School, Quzhou College of Technology, Quzhou, China; ^3^ Department of Natural Medicinal Chemistry, College of Pharmacy, Guangdong Pharmaceutical University, Guangzhou, China

**Keywords:** *Eleutherococcus senticosus* (Rupr. & Maxim.) Maxim. leaves, phenols, saponins, UPLC-MS/MS, *α*-glucosidase inhibitory

## Abstract

*Eleutherococcus senticosus* (Rupr. & Maxim.) Maxim. leaves (ESL) have long been people’s favorite as a natural edible green vegetable, in which phenols and saponins are the main characteristic and bioactive components. This study was first carried out to comprehensively analyze the phenols and saponins in ESL, including phytochemical, qualitative, quantitative, and bioactivity analysis. The results showed that 30 compounds, including 20 phenolic compounds and 7 saponins, were identified. Twelve of them were isolated from *Eleutherococcus* Maxim. for the first time. In the qualitative analysis, 30 phenolic compounds and 28 saponins were accurately detected. Their characteristic cleavage processes were described by UPLC-QTOF-MS/MS. Ten representative ingredients were quantitated in 29 different regions *via* a 4000 QTRAP triple quadrupole tandem mass spectrometer (UPLC-QTRAP-MS/MS), and it was found that S19 (69.89 ± 1.098 mg/g) and S1 (74.28 ± 0.733 mg/g) had the highest contents of total phenols and saponins, respectively. The newly developed analysis method for the quantitative determination was validated for linearity, precision, and limits of detection and quantification, which could be applied to the quality assessment of ESL. *In vitro* experiment, the *α*-glucosidase inhibitory effect of the phenolic fraction was higher than others, indicating that the phenolic content may be related to the hypoglycemic activity. It was also suggested that ESL could be developed as a natural potential effective drug or functional food.

## 1 Introduction

*Eleutherococcus senticosus* (Rupr. & Maxim.) Maxim. (syn. *Acanthopanax senticosus* (Rupr. & Maxim.) Harms, http://www.theplantlist.org), a perennial herb belonging to the Araliaceae family, is mainly distributed in Russia, China, Korea, and Japan, especially in Heilongjiang, Jilin, and Liaoning provinces of the northeast of China (Jia et al., 2021). *Eleutherococcus senticosus* (ES), also called *Acanthopanax senticosus*, *Siberian ginseng*, or *Ciwujia*, known as a famous adaptogen—a herbal medicine that has a non-specific inter-system anti-stress effect throughout the human body (Panossian et al., 2021), first appeared in the Pharmacopoeia of the Union of Soviet Socialist Republics (USSR) as a medicinal plant in 1962. ES was approved by monographed in Russian State Pharmacopoeia (Shikov et al., 2021) and to treat symptoms of asthenia by the European Medicines Agency (EMA), its efficacy has been proved in clinical trials (Gerontakos et al., 2021). According to Chinese Pharmacopoeia, ES is efficient in invigorating the kidney and liver, replenishing the vital essence, and calming the mind (Committee, 2020). Modern pharmacological studies have shown that it tends to stimulate immunity, prevent diseases caused by stress, and treat diseases of the cardio-cerebrovascular system (Kim et al., 2010; Liang et al., 2010; Meng et al., 2018; Xie et al., 2015). While, as a delicious renewable green vegetable and functional tea, ESL is also deeply concerned and loved by the Chinese.

**FIGURE 1:**
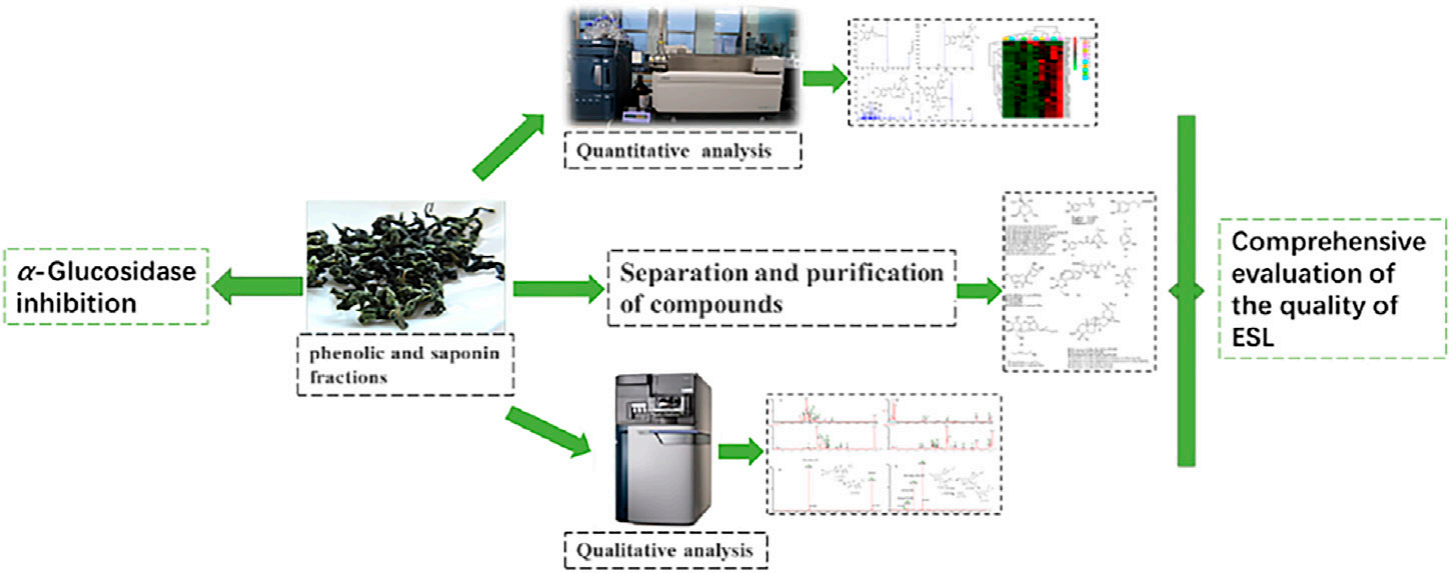
The brief route of experimental research.

Until now, most phytochemical studies have focused on the isolation and identification from ES and demonstrated that it commonly contains saponins, flavonoids, phenylpropanoids, and polysaccharides (Yang et al., 2012; Han et al., 2016; Lau et al., 2019). A few studies have currently been conducted on phenolic constituents and saponins from ESL. The intake of natural phenolic and saponin substances has considerable health benefits, especially for cardiovascular diseases, metabolic diseases, and tumors (Costa et al., 2017; Dong et al., 2019; Singh et al., 2020; Zlab et al., 2020). As the main components in ESL, phenols and saponins possess beneficial effects *in vivo* and in *vitro*, such as anti-cancer (Hayakawa et al., 2020), antiviral (Yan et al., 2018), antibacterial (Lee et al., 2003; Zhou et al., 2017), antioxidative (Tosovic et al., 2017), and inhibitory activities on nitrite production (Lee et al., 2008), and are considered as important active constituents. However, the overall analysis of the composition and content of phenols and saponins in ESL has not been reported. This study mainly investigates the composition analysis, structure cracking law, and quality evaluation of phenols and saponins of ESL (Figure 1).

Due to the diversity, similarity, and complexity of chemical structures, the analysis of compounds becomes a great challenge. Liquid chromatography-electrospray ionization tandem mass spectrometry (LC-MS/MS) is a powerful tool for analyzing compounds (Wang et al., 2016). Because MS can provide the information of molecular formula and fragmentation ions, researchers have identified 42 phenolic compounds in 15 min and 131 ginsenosides in 10 min by an LC-MS/MS method (Wang et al., 2016; Ren et al., 2020), which proved to be an efficient and rapid method for the characterization of compounds. Normally, the long analytical time and low sensitivity are not convenient to rapidly qualify the chemical substances in ESL. To rapidly clarify and analyze the basic chemical substances of ESL, in this study, a new rapid and sensitive ultra-high-performance liquid chromatography-triple quadrupole tandem mass spectrometry (UPLC-MS/MS) method for the thorough detection of major or trace components was firstly established to characterize and quantify phenols and saponins of ESL. Moreover, a total of 30 monomers were isolated, of which 12 compounds (**1–4**, **9**, **10**, **12**, **14**, **19**, **21–23**) were obtained from *Eleutherococcus* Maxim*.* for the first time and 10 compounds, including five phenols and five saponins, were used for quantitative analysis of ESL. This newly developed qualitative and quantitative method based on UPLC-MS/MS could be applied to the quality assessment of ESL. Furthermore, we also firstly compared the *α*-glucosidase inhibitory effect of four different active fractions of ESL *in vitro*. Overall, this study enriches the material basis of ESL to some extent and provides a standard reference for its further rational development and utilization.

## 2 Materials and Methods

### 2.1 Plant Material

The dried ESL were collected in December 2019, Changbai Mountain, Jilin province, and identified by professor Zhenyue Wang of the School of Pharmacy, Chinese Medicine Resources Center, Heilongjiang University of Chinese Medicine.

### 2.2 Standard Samples, Instruments, Chemicals, and Reagents

Ten reference standards of phenols and saponins, including protocatechuic acid (**13**), chlorogenic acid (**7**), methyl 5-*O*-feruloylquinate (**3**), hyperoside (**15**), rutin (**18**), 3-*O*-*α*-*L*-rhamnopyranosyl-(1→2)-*α*-*L*-arabinopyranoside-29-hydroxy oleanolic acid (**24**), 3-*O*-*β*-D-glucopyranosyl-(1→2)-*α*-*L*-arabinopyranoside-29-hydroxy oleanolic acid (**26**), ciwujianoside C4 (**28**), saponin P_E_ (**29**), and ciwujianoside K (**30**) were isolated from ESL in our previous research. Their chemical structures were determined by 1D, 2D NMR spectra, and MS. The purities of all standards were above 98.0%, as elucidated by an HPLC-ELSD method. MS spectra were acquired using a Waters Synapt G2-SI Accurate-Mass Q-TOF (Waters Corp., Milford, MA, United States) and 4000 QTRAP LC/MS system (AB SCIEX, Framingham, United States). ^1^H-NMR (600 MHz) and ^13^C-NMR (150 MHz) data were obtained by a Bruker DPX-600 Spectrometer (Switzerland, Germany). Silica gel (200–300 mesh, Qingdao Haiyang Co., China) and ODS resin (YMC Co., Japan) were used in liquid column chromatography. The preparative HPLC column was a Diamonsil® C18 (5 µm, 10*250 mm) column (DiKMA Co., China). An ACQUITY UPLC HSS T3 column (1.8 μm, 2.1*100 mm, Waters, United States) was used to perform LC-MS analysis. LC-MS grade acetonitrile and formic acid were purchased from Thermo Fisher Scientific (Waltham, United States). Water for UPLC was purified by a Milli-Q water purification system (Darmstadt, Germany). Other reagents and solvents were of analytical grades.

### 2.3 Preparation of Phenolic and Saponin Fractions of *Eleutherococcus Senticosus* (Rupr. & Maxim.) Maxim. Leaves

The dried ESL (5.0 kg) were extracted with 70% ethanol (2 h, three times) under reflux. After recovering ethanol under reduced pressure, the concentrated extract of 2.006 kg (40%) was obtained. The extract was suspended with 12 L water and then successively extracted with petroleum ether and water-saturated n-butanol in a 1:1 volume ratio to obtain the n-butanol extract. The partial n-butanol layer was subjected to PH 2-3 (337.0 g) and eluted by AB-8 macroporous adsorption resin with 30% ethanol solvent to obtain phenolic fraction (96.4 g). In the other part, the saponin fraction (109.1 g) was yielded by elution AB-8 macroporous resin in 60% ethanol.

### 2.4 Isolation and Identification of Chemical Constituents of Phenolic and Saponin Fractions

The above phenolic fraction (61.0 g) was chromatographed on a silica gel (200–300 mesh) column (8*150 cm), with CH_2_Cl_2_-MeOH (10:1, 5:1, 3:1, 2:1, 1:1, 0:1) as the eluent to produce 10 fractions (Fr.1–Fr.10) followed by TLC analysis. Fr.1 was then subjected to a reversed-phase ODS column, eluted with a mixture of MeOH (20–60%). After repeated elution of MeOH (30%, 38%, 40%) in semi-preparative HPLC chromatography, compounds **4** (9.15 mg), **7** (9.61 mg), **12** (8.32 mg), **13** (27.8 mg), and **21** (5.5 mg) were obtained. Compounds **1** (15.84 mg), **2** (80.64 mg), and **3** (33.6 mg) were obtained from Fr.2 by repeated chromatography on Diamonsil^®^ C18 (5 μm, 10*250 mm) column using methanol (30%, 35%) as the eluent. After repeated elution with MeOH (40%), compounds **5** (8.19 mg), **6** (76.92 mg), **8** (11.01 mg), **11** (12.63 mg), and **14** (9.7 mg) obtained from Fr.4. Fr.5, Fr.7, and Fr.8 were purified and recrystallized with MeOH (36%, 40%) to obtain compounds **9** (7.11 mg), **10** (52.69 mg), **15** (23.8 mg), **16** (5.3 mg), and **17** (11.0 mg); compounds **18** (13.35 mg), **19** (10.1 mg), and **20** (7.49 mg); and compounds **22** (3.2 mg) and **23** (5.4 mg). In the same way as above, seven fractions Fr.11–Fr.17 were obtained from the saponin fraction (60.0 g) with TLC identified. The ODS column was applied to elute Fr.15, Fr.16, and Fr.17 with 20%–90% MeOH, further separated and purified by the C18 column, eluted with 70% and 80% MeOH solvent, followed by recrystallization to yield compounds **24** (6.61 mg), **25** (15.0 mg), **26** (11.4 mg), **27** (15.4 mg), **28** (16.7 mg), **29** (9.8 mg), and **30** (12.13 mg). The isolated compounds **1**–**30** were identified by a combination of 1D, 2D-NMR, MS data, and relevant literature.

### 2.5 Sample and Reference Standards Solutions Preparation

ESL from 29 different places was pulverized into powder (40 mesh). The powder (1.0 g) was accurately weighed and suspended in 20 ml MeOH and ultrasonically extracted for 30 min (40 kHz, 500 W, two times). The combined filtrate was evaporated to dryness using a rotary evaporator at 40°C. The residue was dissolved in 5 ml of MeOH. The 10 quantitative reference compounds were dissolved in MeOH and stored at 4°C until analysis. A series of mixed solutions were obtained by diluting the original solutions of these 10 compounds with MeOH. The solutions were prior filtered through a 0.22 μm syringe filter and then quantitatively analyzed.

### 2.6 Qualitative UPLC-QTOF-MS/MS Analysis

An ACQUITY UPLC (Waters, Milford, United States) system in tandem with a QTOF Synapt G2-SI mass spectrometer (Waters, Milford, United States) was acquired for qualitative analysis using an ACQUITY UPLC HSS T3 column (1.8 μm, 2.1*100 mm, Waters, Milford, United States). The chromatography separation was carried out at an ambient temperature of 35°C. The gradient of the eluent mobile phase included acetonitrile with 0.1% formic acid (A), and water with 0.1% formic acid (B): 0–1 min, 2% A; 1–3 min, 2%–10% A; 3–5 min, 10%–20% A; 5–9 min, 20%–55% A; 9–13 min, 55%–70% A; 13–19 min, 70%–80% A; 19–22 min, 80%–98% A; 22–22.5 min, 98%–2% A; 22.5–23 min, 2% A. The flow rate was set at 0.3 ml/min, with a 2 µL injection volume. The MS parameters were optimized as follows: scan type: positive and negative, acquire Mse over the range 100–1,300 Da; scan time: 0.25 s, collision energy: 20–35 V, cone voltage: 40 V.

### 2.7 Quantitation for UPLC-QTRAP-MS/MS Analysis

The quantitation analysis was done *via* an ACQUITY UPLC (Waters Corp., Milford, MA, United States) system tandem 4000 QTRAP mass spectrometer (AB SCIEX, Framingham, United States) with the ACQUITY UPLC HSS T3 column (1.8 μm, 2.1*100 mm, Waters, Milford, United States).

The column temperature was 35°C. The mobile phase was the same as in the qualitative analysis. Isocratic elution was performed as follows: 0–0.5 min, 2%–30% A; 0.5–1 min, 30%–40% A; 1–1.5 min, 40%–55% A; 1.5–3.5 min, 55%–75% A; 3.5–4.5 min, 75%–98% A; 4.5–5.0 min, 98%–98% A. The flow rate was 0.4 ml/min, with 2 µL injection volume. Detection was performed in the negative electrospray ionization mode (ESI^−^) in the Multiple reaction monitoring (MRM). The MS parameters were optimized as follows: TEM: 550°C, curtain gas: 10 psi, IonSpray Voltage: −4500 V, and ion source gas 1 and 2: 55 psi.

### 2.8 *α*-Glucosidase Inhibition Assay

The *α*-glucosidase inhibition activity of all extracts was determined as previously described with minor modifications (Zhang et al., 2017). The solutions of 20 μL different fractions and 60 μL phosphate buffer (67 mM, PH 6.8) containing 20 μL intestinal *α*-glucosidase solution were pre-incubated at 37°C for 5 min. Then, 8 μL PNPG (116 mM) was added to each well. The reaction mixture was incubated at 37°C for 30 min. The absorbance of the mixture was measured at 405 nm before and after incubation. Acarbose was used as the positive control. The inhibitory rate (IR) was calculated as follows:

$$\text{IR}\left(\text{\%}\right)=\left[\left({\text{A}}_{405}{\text{ of control}-\text{A}}_{405}\text{ of samples}\right)/\left({\text{A}}_{405}\text{ of control}\right)\right]\times 100$$

## 3 Results and Discussion

### 3.1 Optimization of the Chromatographic Conditions

**FIGURE 2:**
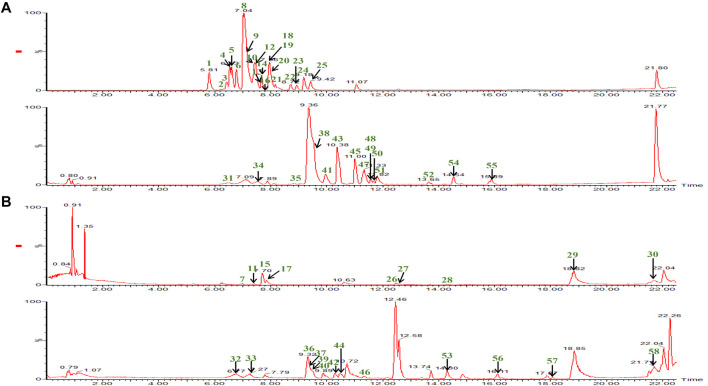
The BPI chromatograms of 58 compounds in phenolic and saponin fractions were detected at positive ion mode **(B)** and negative ion mode **(A)**. The phenolic fraction consists of the upper part of the **(A)** and **(B)** diagram. The remaining part is the saponin fraction.

**FIGURE 3:**
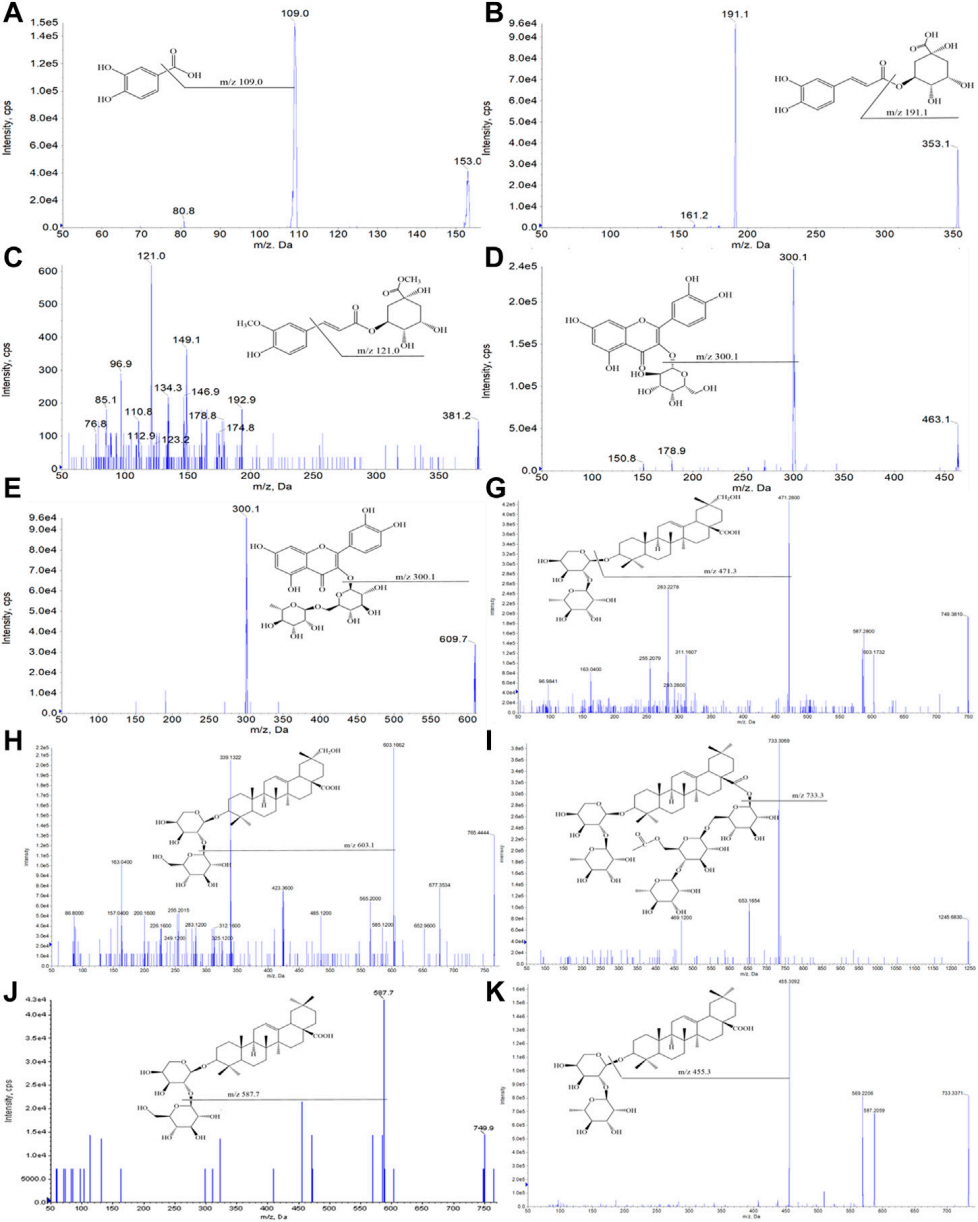
Optimized ion pair diagrams of 10 reference compounds by the 4000 QTRAP mass spectrometry: the process of selecting the best sub-ion (Q3) according to the parent ion (Q1) of the 10 compounds.

In the qualitative and quantitative analysis of ESL, desirable chromatographic separation was yielded by optimizing the column types [Waters ACQUITY UPLC HSS T3 column (1.8 μm, 2.1*100 mm)], the gradient elution procedure, the flow rate (0.3 ml/min and 0.4 ml/min, respectively), and the temperature (35°C). The mobile phase consisting of A (0.1% formic acid aqueous solution) and B (0.1% formic acid acetonitrile solution) was employed to perform gradient elution. All MS spectrometry parameters were optimized to achieve high sensitivity of phenols and saponins. Under the optimized parameters and conditions, 58 compounds were quickly identified (Figure 2). Their characteristic cleavage fragments were shown in Table 1. The optimized parameters, including declustering potential (DP), collision energy (CE) and ion pairs (Figure 3) of the standard compounds in the quantitative analysis were listed in Table 2.

**TABLE 1:** Characterization of compounds in phenolic and saponin fractions by UPLC-MS/MS.

No.	Identification	t_R_ (min)	Characteristic fragment ions	m/z	Formula	Neutral mass
1	Chlorogenic acid	5.81	191.0591[M-Caffeoyl-H]^−^	353.0873[M-H]^−^	C_16_H_18_O_9_	354.12
2	Isochlorogenic acid B	6.21	431.1967 [M-C_4_H_4_O_2_-H]^−^,368.1006[M-C_4_H_4_O_2-_CO_2_-H_2_O-H]^-^,353.0873[M-Caffeoyl-H]^−^,191.9475[M-2Caffeoyl-H]^−^	515.1202[M-H]^−^	C_25_H_24_O_12_	516.11
3	3-*O*-Caffeoylshikimic acid	6.43	179.0363[Caffeic acid-H]^−^,135.0446[Caffeic acid-CO_2_-H]^−^	335.0802 [M-H]^−^	C_16_H_16_O_8_	336.04
4	1,3-Dicaffeoylquinic acid	6.50	335.0802[M-Caffeoyl-H_2_O-H]^−^,191.0530[M-2Caffeoyl-H]^−^,179.0363[Caffeic acid-H]^−^,135.0446[Caffeic acid-CO_2_-H]^−^	515.1202[M-H]^−^	C_25_H_24_O_12_	516.13
5	3,5-Dicaffeoylquinic acid	6.60	335.0802[M-Caffeoyl-H_2_O-H]^-^, 179.0363[Caffeic acid-H]^-^	515.1202[M-H]^−^	C_25_H_24_O_12_	516.12
6	Isochlorogenic acid C	6.78	368.1090[M-C_4_H_4_O_2_-CO_2_-H_2_O-H]^−^,335.0802[M-Caffeoyl-H_2_O-H]^−^,161.0245[Caffeic acid-H_2_O-H]^−^	515.1202[M-H]^−^	C_25_H_24_O_12_	516.13
7	5-*O*-Feruloylquinic acid	6.98	338.3412[M-CO_2_+H]^+^,192.1611[M-Feruloyl+H]^+^,163.0429[Ferulic acid-OCH_3_+H]^+^,103.9565[Coumaic acid-CO_2_-H_2_O +H]^+^	391.0996[M+NA]^+^	C_17_H_20_O_9_	368.11
8	3-*O*-Feruloylquinic acid	7.04	179.0363[Ferulic acid-CH_3_-H]^−^,135.0446[Ferulic acid-CH_3_-CO_2_-H]^−^	367.1090[M-H]^−^	C_17_H_20_O_9_	368.34
9	Rutin	7.20	367.1090[M-Rha-C_2_H_4_O_2_-2H_2_O-H]^−^,301.0327[M-Rha-Glc-H]^−^	609.1498[M-H]^−^	C_27_H_30_O_16_	610.15
10	Hyperoside	7.39	367.1090[M-C_2_H_4_O_2_-2H_2_O-H]^−^,300.0257[M-Gal-2H]^−^,271.0259[M-Gal-CO-2H]^−^	463.0889[M-H]^−^	C_21_H_20_O_12_	464.38
11	5-*O*-Caffeoylshikimic acid	7.40	340.2632[M-H_2_O+Na]^+^,113.9657[M-Caffeoyl-CO_2_-H_2_O+H]^+^	359.2359[M+NA]^+^	C_16_H_16_O_8_	336.04
12	Isoquercitrin	7.46	367.1090[M-C_2_H_4_O_2_-2H_2_O-H]^−^,300.0257[M-Glc-2H]^−^,271.0259[M-Glc-CO-H]^−^	463.0889[M-H]^−^	C_21_H_20_O_12_	464.38
13	Kaempferol-*3*-*O*-robinobioside	7.63	463.0889[M-Rha+H_2_O-H]^−^,285.0412[M-Rha-Gal-H]^−^	593.1528[M-H]^−^	C_27_H_30_O_15_	594.16
14	Kaempferol-*3*-*O*-*α*-L-rhamnopyranosyl(1→2)[*α*-L-rhamnopyranosyl(1→6)]-*β*-glucopyranoside	7.68	721.5026[M-H_2_O-H]^−^,593.1528[M-Rha-H]^−^,367.1006[M-Rha-Glc-C_2_H_4_O-2H_2_O-H]^−^,271.0259[M-Rha-Glc-Rha-CO-H]^−^	739.4935[M-H]^−^	C_33_H_40_O_19_	740.22
15	Syringin	7.70	340.2632[M-OCH_3_+H]^+^,209.1641[M-Glc+H]^+^	395.8013[M+NA]^+^	C_17_H_24_O_9_	372.37
16	Quercitrin	7.79	367.1090[M-C_2_H_4_O-2H_2_O-H]^−^,300.0257[M-Rha-2H]^−^,271.0259[M-Rha-CO-2H]^−^	447.0929[M-H]^−^	C_21_H_20_O_11_	448.34
17	5-*O*-p-Coumaroylquinic acid butyl ester	7.86	396.3058[M+2H]^+^,387.7999[M-H_2_O+H]^+^,113.9657[M-C_4_H_9_-Coumaroyl-CO_2_-H_2_O+H]^+^	417.7812[M+NA]^+^	C_20_H_26_O_8_	394.16
18	1,4-Dicaffeoylquinic acid	7.96	353.0873[M-Caffeoyl-H]^−^,191.0591[M-2Caffeoyl-H]^−^	515.1202[M-H]^−^	C_25_H_24_O_12_	516.13
19	Isorhamnetin-3-*O*-*β*-D-galactopyranoside	7.96	315.0714[M-Gal-H]^−^,284.0337[M-Gal-OCH_3_-H]^−^	477.1127[M-H]^−^	C_22_H_22_O_12_	478.11
20	Kaempferol-7-*O*-*β*-D-glucopyranoside	8.04	300.0287[M-C_5_H_8_O_5_-H]^−^,271.0259[M-C_5_H_8_O_5-_CO-H]^−^	447.1010[M-H]^−^	C_21_H_20_O_11_	448.10
21	Isorhamnetin	8.17	301.0327[M-CH_3_-H]^−^	315.0523[M-H]^−^	C_16_H_12_O_7_	316.06
22	Methyl 1,3-*O*-dicaffeoylquinate	8.72	405.1225[M-C_6_H_5_O_2_-CH_3_-H]^−^,191.9475[M-2Caffeoyl-CH_3_-H]^−^,146.9644[M-2Caffeoyl-CH_3_-CO_2_-H]^−^	529.1407[M-H]^−^	C_26_H_26_O_12_	530.14
23	Ferulic acid	8.93	149.9285[M-CO_2_-H]^−^,133.0272[M-CO_2_-CH_3_-H]^−^	193.0503[M-H]^−^	C_10_H_10_O_4_	194.06
24	Methyl 3,4-*O*-dicaffeoylquinate	9.18	409.1496[M-C_7_H_6_O_2_-H]^−^,367.1006[M-Caffeoyl-H]^−^,146.9644[M-2Caffeoyl-CH_3_-CO_2_-H]^−^	529.1407[M-H]^−^	C_26_H_26_O_12_	530.14
25	Methyl 3,5-*O*-dicaffeoylquinate	9.42	409.1496[M-C_7_H_6_O_2_-H]^−^,191.9475[M-2Caffeoyl-CH_3-_H]^−^,146.9644[M-2Caffeoyl-CH_3_-CO_2_-H]^−^	529.1407[M-H]^−^	C_26_H_26_O_12_	530.14
26	Caffeic acid 3-*O*-glucoside	12.47	299.1089[M-CO_2_+H]^+^	343.2979[M+H]^+^	C_15_H_18_O_9_	342.10
27	Isofraxidin 7-*O*-glucoside	12.63	223.0636[M-Glc+H]^+^	385.3087[M+H]^+^	C_17_H_20_O_10_	384.34
28	3-*O*-p-Coumaroylquinic acid	14.31	303.3065[M-2H_2_O+H]^+^, 113.9657[M-Coumaroyl-CO_2_-H_2_O+H]^+^	339.3412[M+H]^+^	C_16_H_18_O_8_	338.10
29	Quercetin	18.82	282.2811[M-H_2_O+H]^+^	303.1443[M+H]^+^	C_15_H_10_O_7_	302.24
30	Kaempferol 3-*O*-xylopyranosyl-(1→2)-rhamnopyranosyl-(1→6)-glucopyranoside	21.71	579.5396[M-Rha+H]^+^,378.3327[M-Rha-Glc+H]^+^	765.1606[M+K]^+^	C_32_H_38_O_19_	726.20
31	Nipponoside B	6.45	836.5967[M-Rha-C_4_H_7_O_3_-H]^−^,723.5144[M-Rha-Glc+HCOO]^−^	1087.5569[M-H]^−^	C_53_H_84_O_23_	1088.56
32	Silphioside G	6.77	454.8520[M-Glc-GlcA]^+^,396.8013[M-OGlc-OGlcA-CO_2_+H]^+^	816.5898[M+NA]^+^	C_42_H_66_O_14_	793.97
33	Songoroside A	7.27	588.4120[M]^+^,454.3450[M-Xyl]^+^,396.8013[M-OXyl-CO_2_+H]^+^	901.4916[M+NA]^+^	C_35_H_56_O_7_	588.82
34	3*β*-{*O*-*β*-D-Glucopyranosyl-(1→3)-*O*-*β*-D-galactopyranosyl-(1→4)-{*O*-*α*-L-rhamnopyranosyl-(1→2)}c-*O*-*β*-D-glucuronopyranosyl}-16α-hydroxy-13*β*,28-epoxyoleanan	7.56	677.5046[M-Rha-Glc-Gal+HCOO]^−^	1119.5756[M-H]^−^	C_54_H_88_O_24_	1120.43
35	Ciwujianoside D3	8.81	557.1382[M-Rha-GlcAc-Glc-CO_2_-H]^−^,529.1407[M-Rha-GlcAc-Glc-CO_2_-CH_2_O-H]^−^,409.1584[M-OAra-Rha-GlcAc-Glc-CO_2_-H]^−^	1161.5948[M+HCOO]^−^	C_55_H_88_O_23_	1116.57
36	Hederagenin 3-*O*-*β*-D-glucuronopyranosyl methyl ester-28-*O*-*β*-D-glucopyranoside	9.32	635.2262[M-GlcAc+2H]^+^,438.1238[M-OGlcAc-OGlc]^+^	843.3132[M+H]^+^	C_43_H_69_O_16_	842.45
37	Ilexoside XLVIII	9.53	588.4120[M-OGlc-CO_2_]^+^,433.1486[M-OGlcA-Glc-CO_2_+Na]^+^,411.1650[M-OGlcA-OGlc-CO_2_+H]^+^	828.3518[M+H]^+^	C_42_H_67_O_16_	827.96
38	Copteroside B	9.63	409.1584[M-OGlcA-CO_2_-H]^−^,301.0403[M-OGlcA-CO_2_-C_8_H_13_-H]^−^	647.3043[M-H]^−^	C_36_H_56_O_10_	648.12
39	Hederacoside D	9.66	1075.5725[M]^+^,622.2760[M-Ara-Rha-Glc-Glc+H_2_O+H]^+^,433.1486[M-OAra-Rha-Glc-Glc-CO_2_+Na]^+^	1097.5614[M+NA]^+^	C_53_H_86_O_22_	1074.56
40	Ciwujianoside B	9.89	933.4871[M-Rha+K]^+^,423.3284[M-Rha-OAra-Rha-Glc-Glc+H]^+^	1189.6082[M+H]^+^	C_58_H_92_O_25_	1188.36
41	Acanthopanaxoside C	9.95	409.1584[M-GlcA-Ara-CO_2_-H]^−^	763.4387[M-H]^−^	C_41_H_64_O_13_	764.43
42	Oleanolic acid 3-[rhamnosyl-(1→4)-glucosyl-(1→6)-glucoside]	10.10	749.2703[M-OGlcA+H]^+^,455.3599[M-OGlcA-Rha-Glc+H]^+^	949.51217[M+NA]^+^	C_48_H_78_O_17_	926.22
43	Ciwujianoside C1	10.38	941.4941[M-Rha+HCOO] ]^−^,779.4745[M-Rha-Glc+HCOO]^−^,571.3712[M-Rha-Glc-Glc-H]^−^,391.1456[M-Rha-Glc-Glc-Ara-CO_2_-H]^−^	1087.5669[M+HCOO]^−^	C_52_H_82_O_21_	1042.53
44	Hederagenin 28-O-*β*-D-glucopyranoside	10.49	439.3583[M-Glc-CH_2_OH]^+^,423.3284[M-OGlc-CH_2_OH]^+^	635.7875[M+H]^+^	C_36_H_58_O_9_	634.14
45	3*β*-{O-*α*-L-Rhamnopyranosyl-(1→4)-O-*α*-L-rhamnopyranosyl-(1→4)-[O-*α*-L-rhamnopyranosyl-(1→2)]-O-*β*-D-glucopyranosyl-(1→x)-O-*β*-D-glucuronopyranosyl}-16*α*-hydroxy-13*β*,28-epoxyoleanane	11.00	571.3712[M-Glc-2Rha-ORha-CO_2_-H]^−^	1197.5474[M-H]^−^	C_57_H_98_O_26_	1198.63
46	Silphioside F	11.31	555.1868[M-C_2_H_4_O_2_-H_2_O+H]^+^,393.1572[M-OGlcA-CO_2_]^+^	655.3060[M+NA]^+^	C_36_H_56_O_9_	632.80
47	Ciwujianoside D2	11.33	571.1937[M-Rha-GlcAc-Glc-H]^−^	1129.5637[M+HCOO]^−^	C_54_H_84_O_22_	1084.55
48	3-*O*-*β*-D-Glucopyranoside-29-hydroxy oleanolic acid	11.57	603.3969[M-CO_2_-H]^−^,571.1937[M-CO_2_-CH_2_OH-H]^−^	649.4061[M-H]^−^	C_45_H_78_O_2_	650.34
49	Eleutheroside K	11.59	649.4061[M-ORha+HCOO]^−^,603.3969[M-Rha+H_2_O-2H]^−^,571.1937[M-ORha-H]^−^	733.3565 [M-H]^−^	C_41_H_66_O_11_	734.45
50	Acanthopanaxoside E	11.68	603.3969[M-Glc-CO_2_-H]^−^,587.4081[M-OGlc-CO_2_-H]^−^	809.4473[M-H]^−^	C_42_H_66_O_15_	810.56
51	Ciwujianoside D1	11.82	603.3969[M-Rha-GlcAc-Glc+H_2_O-2H]^−^,571.1937[M-Rha-GlcAc-OGlc-H]^−^	1145.5898[M+HCOO]^−^	C_55_H_88_O_22_	1100.58
52	Eleutheroside I	13.65	733.4275[M-H]^−^,571.3712[M-ORha-H]^−^	779.4380[M+HCOO]^−^	C_41_H_66_O_11_	734.45
53	*β*-Sitosterol	14.30	346.3379[M-C_5_H_10_+2H]^+^,302.3113[M-C_8_H_16_]^+^,113.9657[M-C_21_H_32_O+H]^+^	437.2013[M+NA]^+^	C_29_H_50_O	414.71
54	Ciwujianoside E	14.54	717.4304[M-H]^−^,571.3712[M-Rha-H]^−^	763.4387[M+HCOO]^−^	C_40_H_62_O_11_	718.91
55	30-Norolean-12,20(29)-dien-28-oic acid-3-*O*-*β*-D-glucopyranosyl-(1→2)-*α*-L-arabinopyranoside	15.89	733.4630[M-H]^−^,571.3712[M-Glc-H]^−^	779.4775[M+HCOO]^−^	C_41_H_66_O_11_	734.45
56	3-*O*-*α*-L-Arabinopyranoside oleanolic acid	16.11	437.1922[M-Ara-H_2_O+H]^+,^396.3492[M-OAra-CO_2_+H]^+^	589.4186[M+H]^+^	C_35_H_56_O_7_	588.40
57	Daucosterol	18.04	577.1353[M+H]^+^,338.3492[M-Glc-C_7_H_14_+H]^+^,301.1434[M-Glc-C_8_H_16_]^+^	599.1245[M+NA]^+^	C_35_H_60_O_6_	576.85
58	Hederagenin 3-*O*-*β*-D-glucuronopyranoside-6-*O*-methyl ester	21.71	379.3438[M-OGlcAc-CO_2_-CH_2_OH]^+^	685.4426[M+NA]^+^	C_37_H_58_O_10_	662.40

**TABLE 2:** The selective ion-pair, DP, and CE of 10 reference compounds.

No.	Compounds	Q1 [M-H]^−^	Q3	DP/V	CE/V
A	Protocatechuic acid	153.0	109.0	−80.04	−23.74
B	Chlorogenic acid	353.1	191.1	−80.59	−19.81
C	Methyl 5-*O*-feruloylquinate	381.2	121.0	−107.28	−37.45
D	Hyperoside	463.1	300.1	−144.10	−38.99
E	Rutin	609.7	300.1	−188.87	−55.38
G	3-*O*-*α*-L-Rhamnopyranosyl-(1→2)-*α*-L-arabinopyranoside-29-hydroxy oleanolic acid	749.4	471.3	−205.60	−59.14
H	3-*O*-*β*-D-Glucopyranosyl-(1→2)-*α*-L-arabinopyranoside-29-hydroxy oleanolic acid	765.4	603.1	−212.51	−58.04
I	Ciwujianoside C4	1,245.6	733.3	−171.82	−79.83
J	Saponin P_E_	749.9	587.7	−217.18	−59.35
K	Ciwujianoside K	733.5	455.3	−191.59	−61.10

### 3.2 Isolation of Compounds

#### 3.2.1 Isolation of Chemical Constituents

**FIGURE 4:**
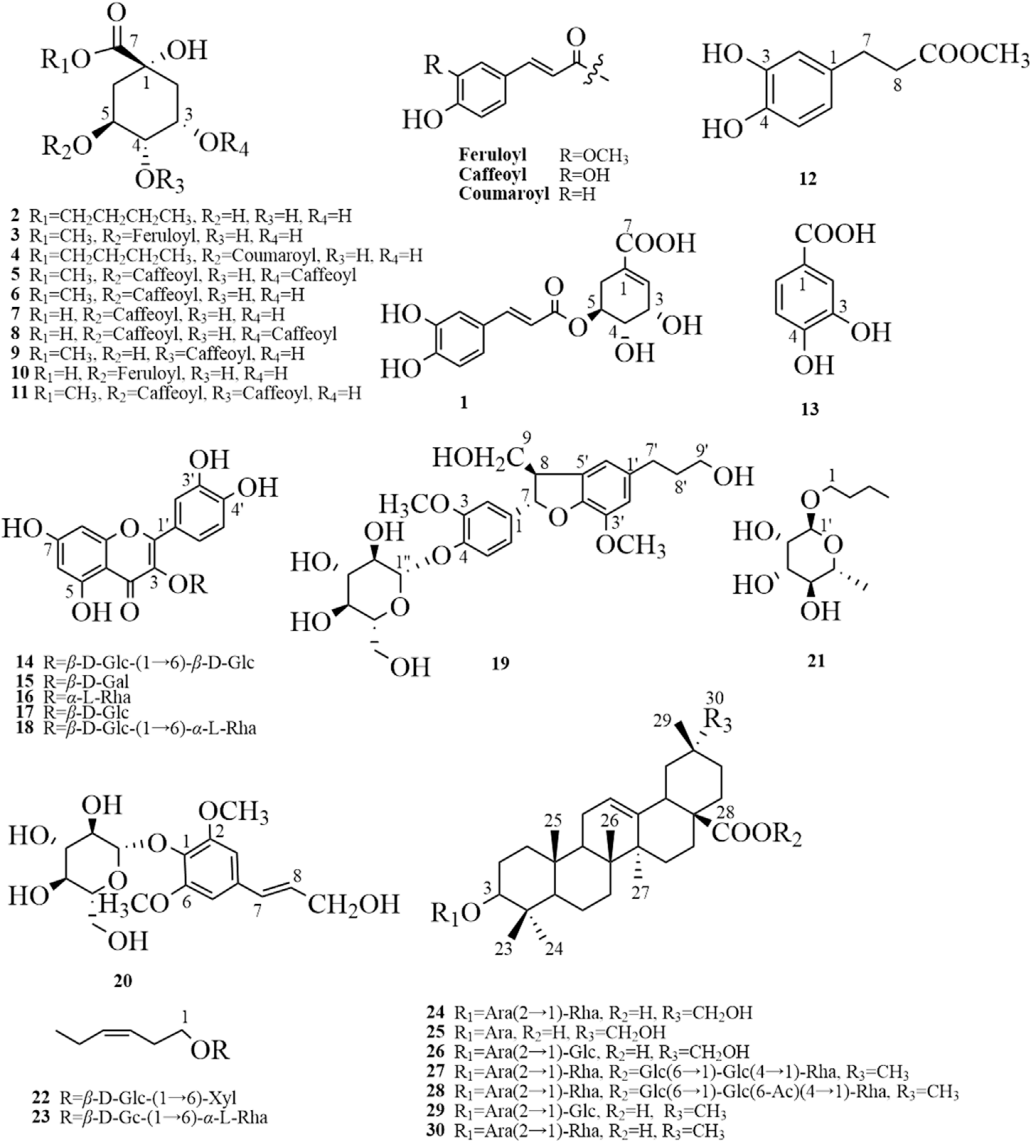
The chemical structures of 30 compounds were isolated and identified from phenolic and saponin fractions. 5-*O*-Caffeoylshikimic acid (**1**), quinic acid butyl ester (**2**), methyl 5-*O*-feruloylquinate (**3**), 5-*O*-*p*-coumaroylquinic acid butyl ester (**4**), methyl 3,5-di-*O*-caffeoyl quinate (**5**), methyl chlorogenate (**6**), chlorogenic acid (**7**), 3,5-di-*O*-caffeoylquinic acid (**8**), 4-*O*-caffeoylquinic acid methyl ester (**9**), 5-*O*-feruloylquinic acid (**10**), methyl 3,4-di-*O*-caffeoyl quinate (**11**), 3,4-dihydroxybenzenepropionic acid methyl ester (**12**), protocatechuic acid (**13**), quercetin 3-*O*-*β*-D-glucopyranosyl-(1→6)-*β*-D-glucopyranoside (**14**), hyperoside (**15**), quercitrin (**16**), quercetin 3-*O*-*β*-D-glucopyranoside (**17**), rutin (**18**), (7S, 8R)-urolignoside (**19**), syringin (**20**), n-butyl-1-*O*-*α*-L-rhamnopyranoside (**21**), (Z)-Hex-3-en-1-ol *O*-*β*-D-xylopyranosyl-(1″-6′)-*β*-D-glucopyranoside (**22**), hexenyl-rutinoside (**23**), 3-*O*-*β*-L-rhamnopyranosyl-(1→2)-*α*-L-arabinopyranoside-29-hydroxy oleanolic acid (**24**), 3-*O*-*α*-arabinopyranoside 29-hydroxy oleanolic acid (**25**), 3-*O*-*α*-D-glucopyranosyl-(1→2)-*α*-L-arabinopyranoside-29-hydroxy oleanolic acid (**26**), hederasaponin B (**27**), ciwujianoside C4 (**28**), saponin P_E_ (**29**), and ciwujianoside K **(30**).

Thirty compounds (chemical structures shown in Figure 4) include 20 phenols, 7 saponins, and 3 glycosides, of which 12 compounds (**1–4**, **9**, **10**, **12**, **14**, **19**, **21–23**) were isolated from *Eleutherococcus* Maxim. for the first time using a combination of chromatographic methods. The compounds were identified based on extensive NMR and MS data and comparison to published literature data when available.

#### 3.2.2 Identification of Chemical Constituents

The isolated compounds **1**–**30** (chemical structures shown in Figure 4) were identified by a combination of 1D, 2D-NMR, and MS data. Compounds **1–4**, **9**, **10**, **12**, **14**, **19**, and **21–23** were obtained from *Eleutherococcus* Maxim. for the first time, and their NMR data were provided here.

##### 3.2.2.1 5-*O*-Caffeoylshikimic Acid (**1**)

Yellow amorphous powder: (−) HR-ESI-MS, m/z 335.0748 [M-H]^−^, calculated for molecular formula C_16_H_16_O_8_. ^1^H NMR (600 MHz, CD_3_OD): δ 7.56 (1H, d, *J* = 15.9 Hz,H-7′), 7.04 (1H, d, *J* = 2.1 Hz, H-2′), 6.95 (1H, dd, *J* = 8.2, 2.1 Hz, H-6′), 6.86 (1H, brs, H-2), 6.78 (1H, d, *J* = 8.2 Hz, H-5′), 6.28 (1H, d, *J* = 15.9 Hz, H-8′), 5.25 (1H m, H-5), 4.41(1H, brs, H-3), 2.86 (1H, dd, *J* = 18.4, 5.2 Hz, H-6*α*), 2.32 (1H, dd, J = 18.4, 5.5 Hz, H-6*β*). ^13^C-NMR (150 MHz, CD_3_OD): δ 130.3 (C-1), 139.0 (C-2), 67.3 (C-3), 70.0 (C-4), 71.4 (C-5), 29.2 (C-6), 169.7 (C-7), 127.7 (C-1′), 115.2 (C-2′), 146.8 (C-3′), 147.3 (C-4′), 116.5 (C-5′), 123.1 (C-6′), 149.7 (C-7′), 115.1 (C-8′), 168.7 (C-9′). The NMR data were consistent with the literature (Wan et al., 2012).

##### 3.2.2.2 Quinic Acid Butyl Ester (**2**)

Yellowish amorphous powder: (+) HR-ESI-MS, m/z 248.9876 [M]^+^, calculated for molecular formula C_11_H_20_O_6_. ^1^H NMR (600 MHz, CD_3_OD): δ 2.08 (2H, m, H-2), 4.09 (1H, m, H-3), 3.40(1H, dd, *J* = 8.8, 3.2 Hz, H-4), 3.99 (1H, m, H-5), 1.85(2H, m, H-6), 4.15 (2H, m, H-8), 1.65(2H, m, H-9), 1.41(2H, m, H-10), 0.96 (3H, t, *J* = 7.4 Hz, H-11). ^13^C-NMR (150 MHz, CD_3_OD): δ 76.9 (C-1), 38.3 (C-2), 71.6 (C-3), 76.8 (C-4), 68.1 (C-5), 42.2 (C-6), 175.6 (C-7), 66.3 (C-8), 31.7 (C-9), 20.1 (C-10), 14.0 (C-11). The NMR data were consistent with the literature (Shi et al., 2014).

##### 3.2.2.3 Methyl 5-*O*-Feruloylquinate (**3**)

Yellow amorphous powder: (−) HR-ESI-MS, m/z 381.2299 [M-H]^−^, calculated for molecular formula C_18_H_22_O_9_. ^1^H NMR (600 MHz, CD_3_OD): *δ* 2.02,2.12 (each 1H, m, H-2*α*,*β*), 4.14(1H, m, H-3), 3.41(1H, dd, *J* = 8.6,3.2 Hz, H-4), 5.28(1H, m, H-5), 2.09,2.19(each 1H, m, H-6*α*, *β*), 4.03 (3H, s, 7-OCH_3_), 7.04(1H, d, *J* = 2.1 Hz, H-2′), 3.69(3H, s, 3′-OCH_3_), 6.78 (1H, d, *J* = 8.1 Hz, H-5′), 6.95 (1H, dd, *J* = 8.2, 2.1 Hz, H-6′), 6.22 (1H, d, *J* = 15.9 Hz, H-7′), 7.53(1H, d, *J* = 15.9 Hz, H-8′). ^13^C-NMR (150 MHz, CD_3_OD): δ 68.2 (C-1), 38.3 (C-2), 76.8 (C-3), 76.6 (C-4), 72.1 (C-5), 38.1 (C-6), 176.0 (C-7), 58.4 (7-OCH3), 123.0 (C-1′), 116.6 (C-2′), 147.2 (C-3′), 52.4 (3′-OCH_3_), 146.9 (C-4′), 115.1 (C-5′), 127.7 (C-6′), 149.7 (C-7′), 112.8 (C-8′), 168.3 (C-9′). The NMR data were consistent with the literature (Ida et al., 1993).

##### 3.2.2.4 5-*O*-*p*-Coumaroylquinic Acid Butyl Ester (**4**)

Light brown amorphous powder: (+) HR-ESI-MS, m/z 789.6702 [2M+H]^+^, calculated for molecular formula C_20_H_26_O_8_. ^1^H NMR (600 MHz, CD_3_OD): δ 2.20(2H, m, H-2*α*, *β*), 5.28(1H, m, H-3), 3.73(1H, dd, *J* = 7.5, 3.2 Hz, H-4),4.15(1H, m, H-5), 2.01 (1H, dd, *J* = 13.3, 6.5 Hz, H-6*α*), 2.20 (1H, dd, *J* = 13.7, 3.3 Hz, H-6*β*), 7.46(1H, d, *J* = 8.6 Hz, H-2′), 6.81(1H, d, *J* = 8.6 Hz, H-3′), 6.81 (1H, d, *J* = 8.6 Hz, H-5′), 7.46(1H, d, *J* = 8.6 Hz, H-6′), 7.60 (1H, d, *J* = 16.0 Hz, H-7′), 6.29(1H, d, *J* = 15.9 Hz, H-8′), 4.12(2H, m, H-1″), 1.64(2H, m, H-2″), 1.41(2H, m, H-3″), 0.95(3H,t,*J* = 7.4 Hz, H-4″). ^13^C-NMR (150 MHz, CD_3_OD): δ 75.9 (C-1), 35.8 (C-2), 72.2 (C-3), 72.7 (C-4), 70.4 (C-5), 38.1 (C-6), 175.5 (C-7), 127.1 (C-1′), 131.2 (C-2′), 115.9 (C-3′), 161.4 (C-4′), 115.9 (C-5′), 131.2 (C-6′), 145.8 (C-7′), 115.2 (C-8′), 168.3 (C-9′), 66.1 (C-1″), 31.7 (C-2″), 20.1 (C-3″), 14.0 (C-4″). The NMR data were consistent with the literature (Osawa et al., 2001).

##### 3.2.2.5 4-*O*-Caffeoylquinic Acid Methyl Ester (**9**)

White amorphous powder: (+) HR-ESI-MS, m/z 391.1035 [M+Na]^+^, calculated for molecular formula C_17_H_20_O_9_. ^1^H NMR (600 MHz, CD_3_OD): *δ* 2.07, 2.20 (each 1H, m, H-2*α*, *β*), 4.29 (1H, m, H-3), 4.82 (1H, m, H-4), 4.25 (1H, m, H-5), 2.03, 2.19 (each 1H, H-6*α*, *β*), 7.63 (1H,d, *J* = 15.9 Hz, H-7), 7.07 (1H, d, *J* = 2.1 Hz, H-2′), 6.78 (1H, d, *J* = 8.2 Hz, H-5′), 6.97 (1H, dd, *J* = 8.3, 2.1 Hz, H-6′), 7.63 (1H, d, *J* = 15.9 Hz, H-7′), 6.36 (1H, d, *J* = 15.9 Hz, H-8′), 3.75 (3H, s, 7-OCH_3_). ^13^C-NMR (150 MHz, CD_3_OD): δ 76.5 (C-1), 42.2 (C-2), 69.1 (C-3), 78.6 (C-4), 65.7 (C-5), 38.5 (C-6), 175.7 (C-7), 53.0 (7-OCH3), 127.9 (C-1′), 115.2 (C-2′), 146.9 (C-3′), 149.6 (C-4′), 116.5 (C-5′), 123.0 (C-6′), 147.2 (C-7′), 115.4 (C-8′), 169.0 (C-9′). The NMR data were consistent with the literature (Chen et al., 2016).

##### 3.2.2.6 5-*O*-Feruloylquinic Acid (**10**)

White amorphous powder: (+) HR-ESI-MS, m/z 391.1035 [M+Na]^+^, calculated for molecular formula C_17_H_20_O_9_. ^1^H NMR (600 MHz, CD_3_OD): δ 2.12 (4H, m, H-2, 6), 4.14 (1H, m, H-3), 3.73 (1H, dd, *J* = 7.5, 3.1 Hz, H-4), 5.28 (1H, m, H-5), 7.04 (1H, d, *J* = 2.1 Hz, H-2′), 6.78 (1H, d, *J* = 8.1 Hz, H-5′), 6.95 (1H, dd, *J* = 8.2, 2.1 Hz, H-6′), 7.53 (1H, d, *J* = 15.9 Hz, H-7′), 6.22 (1H, d, *J* = 15.9 Hz, H-8′), 3.69 (3H, s, 3′-OCH_3_). ^13^C-NMR (150 MHz, CD_3_OD): δ 75.8 (C-1), 38.0 (C-2), 72.1 (C-3), 70.3 (C-4), 72.6 (C-5), 37.8 (C-6), 175.5 (C-7), 53.0 (7-OCH3), 127.7 (C-1′), 115.2 (C-2′), 149.7 (C-3′), 146.9 (C-4′), 116.6 (C-5′), 123.0 (C-6′), 147.2 (C-7′), 115.1 (C-8′), 168.3 (C-9′). The NMR data were consistent with the literature (Menozzi-Smarrito et al., 2011).

##### 3.2.2.7 3,4-Dihydroxybenzenepropionic Acid Methyl Ester (**12**)

Yellow oily matter: (−) HR-ESI-MS, m/z 195.0631 [M-H]^−^, calculated for molecular formula C_10_H_12_O_4_. ^1^H NMR (600 MHz, CD_3_OD): δ 6.62 (1H, d, *J* = 2.1 Hz, H-2), 6.66 (1H, d, *J* = 8.0 Hz, H-5), 6.50 (1H, dd, *J* = 8.0, 2.1 Hz, H-6), 2.55 (2H, t, *J* = 7.6 Hz, H-7), 2.76 (2H, t, *J* = 7.6 Hz, H-8), 3.63 (3H, s, 9-OCH_3_). ^13^C-NMR (150 MHz, CD_3_OD): δ 133.5 (C-1), 116.4 (C-2), 144.7 (C-3), 146.2 (C-4), 116.4 (C-5), 120.5 (C-6), 31.4 (C-7), 37.1 (C-8), 175.4 (C-9), 52.0 (9-OCH_3_). The NMR data were consistent with the literature (Meng et al., 2014).

##### 3.2.2.8 Quercetin 3-*O*-β-D-Glucopyranosyl-(1→6)-β-D-glucopyranoside (**14**)

White amorphous powder: (+) HR-ESI-MS, m/z 627.5396 [M+H]^+^, calculated for molecular formula C_27_H_30_O_17_.^1^H NMR (600 MHz, CD_3_OD): δ 6.21 (1H, d, *J* = 2.1 Hz, H-6), 6.40 (1H, d, *J* = 2.1 Hz, H-8), 7.84 (1H, d, *J* = 2.2 Hz, H-2′), 6.87 (1H, d, *J* = 8.5 Hz, H-5′), 7.59 (1H, dd, *J* = 8.5, 2.2 Hz, H-6′), 5.25 (1H, d, *J* = 7.7 Hz, H-1″), 5.17 (1H, d, *J* = 7.8 Hz, H-1‴). ^13^C-NMR (150 MHz, CD_3_OD): δ 158.5 (C-2), 135.8 (C-3), 179.6 (C-4), 163.1 (C-5), 99.9 (C-6), 166.1 (C-7), 94.7 (C-8), 158.8(C-9), 105.6 (C-10),123.0 (C-1′), 117.8 (C-2′), 145.8 (C-3′), 150.0 (C-4′), 116.1 (C-5′), 123.2 (C-6′), 105.4 (C-1″), 75.8 (C-2″), 78.4 (C-3″), 73.2 (C-4″), 77.2 (C-5″), 70.1 (C-6″), 104.3 (C-1‴), 75.1 (C-2‴), 77.2 (C-3‴), 71.3 (C-4‴), 78.2 (C-5‴), 62.0 (C-6‴). The NMR data were consistent with the literature (Zeng et al., 2020).

##### 3.2.2.9 (7S, 8R)-Urolignoside (**19**)

White amorphous powder: (+) HR-ESI-MS, m/z 545.1989 [M+Na]^+^, calculated for molecular formula C_26_H_34_O_11_. ^1^H NMR (600 MHz, CD_3_OD): δ 7.03 (1H, d, *J* = 1.4 Hz, H-2), 3.86 (3H, s, 3- OCH_3_), 7.14 (1H, d, *J* = 8.4 Hz, H-5), 6.93 (1H, dd, *J* = 8.4, 2.0 Hz, H-6), 5.56 (1H, d, *J* = 5.9 Hz, H-7), 3.45 (1H, m, H-8), 3.68, 3.76 (each 1H, H-9*α*, *β*), 6.72 (1H, brs, H-2′), 3.83 (3H, s, 3′-OCH_3_), 6.74 (1H, brs, H-6′), 2.63 (2H, *t*, *J* = 7.5 Hz, H-7′), 1.82 (2H, m, H-8′), 3.57 (2H, *t*, *J* = 6.5 Hz, H-9′), 4.89 (1H, d, *J* = 7.4 Hz, H-1″). ^13^C-NMR (150 MHz, CD_3_OD): *δ* 138.4(C-1), 111.2 (C-2), 151.0 (C-3), 56.8 (3-OCH_3_), 147.1 (C-4), 116.2 (C-5), 119.4 (C-6), 88.5 (C-7), 55.7 (C-8), 65.1 (C-9), 137.1 (C-1′), 114.2 (C-2′), 143.5 (C-3′), 56.7(3′-OCH_3_), 145.3 (C-4′), 129.6 (C-5′), 118.0 (C-6′), 32.9 (C-7′), 35.8 (C-8′), 62.3 (C-9′), 102.8 (C-1″), 74.9 (C-2″), 77.9 (C-3″), 71.4 (C-4″), 78.2 (C-5″), 62.5 (C-6″). The NMR data were consistent with the literature (Zhao et al., 2018).

##### 3.2.2.10 N-Butyl-1-*O*-α-L-Rhamnopyranoside (**21**)

Colorless oily matter: (−) HR-ESI-MS, m/z 255.8209[M+2H_2_O-H]^−^, calculated for molecular formula C_10_H_20_O_5_. 1H, m, NMR (600 MHz, CD_3_OD): δ 4.65 (1H, brs, H-1), 3.77 (1H, dd, *J* = 3.4 Hz, H-2), 3.63 (1H, dd, *J* = 1.7 Hz, H-3), 3.31 (H-4), 3.57 (1H, m, H-5), 1.26 (3H, d, *J* = 6.3 Hz, H-6) 3.39, 3.67 (each 1H, m, H-1′), 1.57 (2H, m, H-2′), 1.41 (2H, m, H-3′), 0.94 (3H, t, *J* = 7.4 Hz, H-4′). ^13^C-NMR (150 MHz, CD_3_OD): δ 101.7 (C-1), 72.5 (C-2), 74.0 (C-3), 72.4 (C-4), 69.8 (C-5), 18.0 (C-6), 68.3 (C-1′), 32.8 (C-2′), 20.5 (C-3′), 14.2 (C-4′). The NMR data were consistent with the literature (Mallavadhani and Narasimhan, 2009).

##### 3.2.2.11 (Z)-Hex-3-en-1-ol O-β-D-Xylopyranosyl-(1″-6′)-β-D-Glucopyranoside (**22**)

White amorphous powder; (+) HR-ESI-MS, m/z 417.1705 [M+Na]^+^, calculated for molecular formula C_17_H_30_O_10_.^1^H NMR (600 MHz, CD_3_OD): *δ* 3.55,3.83 (each 1H, m, H-1), 2.38 (2H, q, *J* = 7.2 Hz, H-2), 5.39 (1H, dtt, *J* = 10.8, 5.1, 1.4 Hz, H-3), 5.45 (1H, dtt, *J* = 12.1, 6.9, 1.4 Hz, H-4), 2.08 (2H, qd, *J* = 7.4, 1.4 Hz, H-5), 0.97 (3H, t, *J* = 7.6 Hz, H-6) 4.32 (1H, d, *J* = 7.5 Hz, H-1′), 4.08 (1H, dd, *J* = 11.5, 2.1 Hz, H-6′*α*), 3.74 (1H, dd, *J* = 11.5, 5.6 Hz, H-6′*β*), 4.27 (1H, d, *J* = 7.8 Hz, H-1″). ^13^C-NMR (150 MHz, CD_3_OD): δ 70.7 (C-1), 28.8 (C-2), 125.9 (C-3), 134.5 (C-4), 21.6 (C-5), 14.7 (C-6), 104.4 (C-1′), 74.9 (C-2′), 78.0 (C-3′), 71.2 (C-4′), 77.7 (C-5′), 69.8 (C-6′), 105.5 (C-1″), 75.1 (C-2″), 77.0 (C-3″), 71.5 (C-4″), 67.0 (C-5″). The NMR data were consistent with the literature (Iha et al., 2012).

##### 3.2.2.12 Hexenyl-Rutinoside (**23**)

White amorphous powder: (+) HR-ESI-MS, m/z 408.3348 [M]^+^, calculated for molecular formula C_18_H_32_O_10_. ^1^H NMR (600 MHz, CD_3_OD): δ 3.54,3.83 (each 1H, m, H-1), 2.39 (2H, m, H-2), 5.39 (1H, m, H-3), 5.46 (1H, m, H-4), 2.08 (2H, m, H-5), 0.97 (3H, t, *J* = 7.8 Hz, H-6) 4.25 (1H, d, *J* = 7.5 Hz, H-1′), 4.74 (1H, d, *J* = 1.7 Hz, H-1″), 1.26 (3H, d, *J* = 6.2 Hz, H-6″). ^13^C-NMR (150 MHz, CD_3_OD): δ 70.6 (C-1), 28.9 (C-2), 125.9 (C-3), 134.6 (C-4), 21.6 (C-5), 14.7 (C-6), 104.5 (C-1′), 75.1 (C-2′), 78.1 (C-3′), 71.7 (C-4′), 76.9 (C-5′), 68.1 (C-6′), 102.3 (C-1″), 72.6 (C-2″), 73.6 (C-3″), 74.1 (C-4″), 69.8 (C-5″), 18.1 (C-6″). The NMR data were consistent with the literature (Kil et al., 2019).

### 3.3 Identity Assignment and Confirmation of the Phenolic Compounds and Saponins in ESL

In the present study, the phenolic and saponin fractions of ESL extracted in Method 2.3 were used as the representative sample for qualitative analysis because of its most comprehensive phenolic and saponins profile. According to the accurate fragmentation law of fragment ions and the literature, 58 compounds (Figure 2; Table 1), including 30 phenols and 28 saponins, were identified. All compounds were divided into three categories: phenolic acids, flavonoids, and saponins.

#### 3.3.1 Phenolic Compounds

**FIGURE 5:**
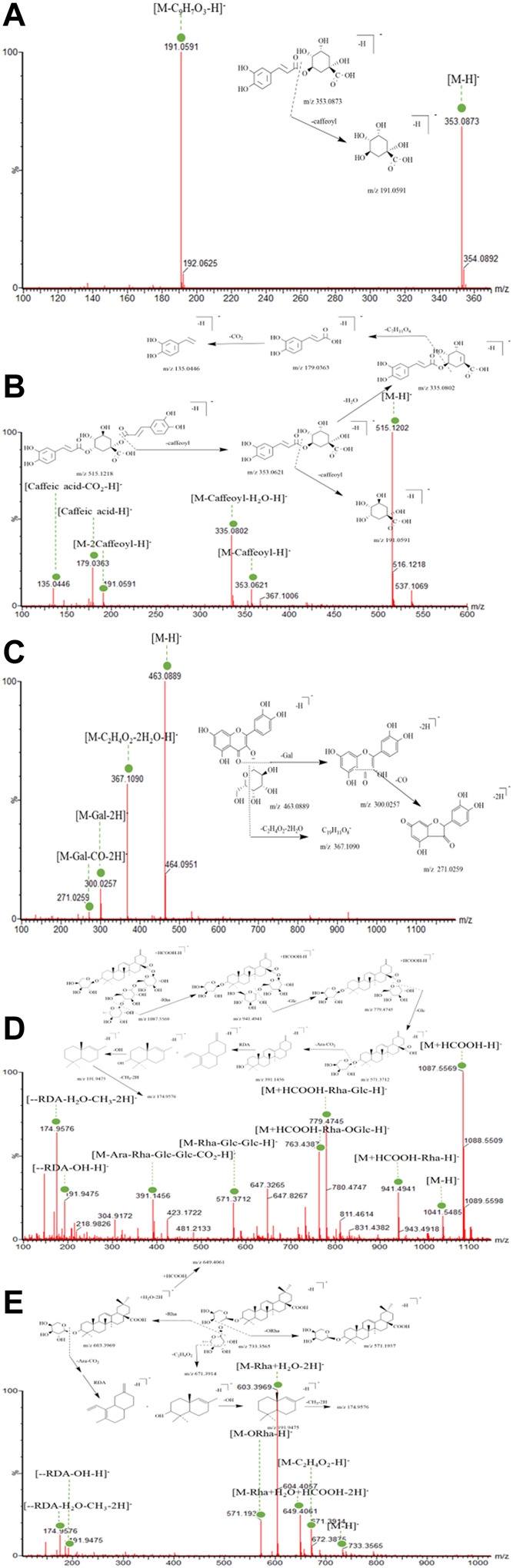
UPLC-QTOF-MS/MS spectra and the cleavage pathways of chlorogenic acid **(A)**, 1,3-dicaffeoylquinic acid **(B)**, hyperoside **(C)**, ciwujianoside C1 **(D),** and eleutheroside K **(E)**.

To date, phenols in ESL have not been systematically characterized through UPLC-MS/MS. As shown in Table 1, 30 phenolic compounds were identified, mainly divided into phenolic acids and flavonoids based on their structural characteristics. Among them, most of these phenolic acids were composed of one or two caffeic acids and one quinic acid or their derivatives by dehydration condensation, such as compounds **1–8**, **11**, **17**, **18**, **22–26**, and **28**. Generally, due to the readily dissociated ester bond, these phenolic acids were inclined to lose quinic acid moieties (156 Da), caffeoyl (162 Da), feruloyl (176 Da), or coumaroyl moieties (146 Da) in the MS spectra as one characteristic of them. Moreover, the further CO_2_ (44 Da) and OCH_3_/CH_3_ (31/15 Da) loss were other characteristic fragmentation behavior of compounds **2–8**, **11**, **17**, **22–26**, and **28** because of the oxygen methyl or carboxyl group in their structures (Ren et al., 2020). For example, the 191 Da, 192 Da, 113 Da, 179 Da, 353 Da, and 367 Da fragment ions in compounds **1–5**, **7**, **8**, **17**, **18**, **24**, and **28** were caused by the loss of caffeoyl, feruloyl, coumaroyl, and/or quinic acid ion. Fragment 135 Da, 338 Da, 179 Da, 299 Da, 149 Da, 163 Da, and 103 Da in compounds **3–4**, **8**, **26**, **23**, and **7** were typically obtained by direct loss of CO_2_ or OCH_3_/CH_3_ fragment ions. Chlorogenic acid (**1**, Figure 5A) and 1,3-dicaffeoylquinic acid (**4**, Figure 5B) were used as representative phenolic acids to clarify the unique fragmentation pathway in this study.

Among the 11 flavonoids rapidly identified (Table 1), all but two flavonoid aglycones belong to the *O*-glycosyl type. For flavonoid *O*-glycoside, the most typical fragmentation behavior was C-*O* bond cleavage, which frequently produced a glycosyl moiety (Huang et al., 2015). In addition, all flavonoids had the same mother nucleus, which was quercetin (302 Da), kaempferol (286 Da), and rhamnetin (316 Da), respectively. Accordingly, compounds **9**, **10**, **12**, and **16** caused the parent nuclear fragment ion 301/300 Da [quercetin-H/2H]^−^ due to the loss of rutinose (308 Da), galactose (162 Da), glucose (162 Da), and rhamnoside (146 Da). The representative fragments [M-H-146–162]^−^ in compound **13** was [kaempferol-H]^−^, which were obtained by losing one robinobioside (308 Da) group. Similarly, compound **19** lost one galactose (162 Da) to produce fragmentation 315 Da [rhamnetin-H/M-H-162]^−^. Flavonoid *O*-glycosides, such as compounds **9**, **10**, and **12**, often showed the loss of C_2_H_4_O_2_ (60 Da), attributed to the split through the sugar moiety. Moreover, the loss of partial division of sugar unit has also been found in some compounds (**14**, **16**, **20**) of this type, which might be a characteristic fragmentation behavior of these compounds with splitting through the glycosyl. Due to the reverse Diels–Alder reaction (RDA), flavonoids were trend to produce characteristic fragmentation (CO, 28 Da) behavior (Ren et al., 2020; L.; Xie et al., 2019). Hyperoside (**10**, Figure 5C) was used as the example of flavonoid *O*-glycosides and flavones, respectively, to illuminate their characteristic fragmentation pathway.

#### 3.3.2 Saponins

According to the fragmentation features and combined with literature verification (Li et al., 2010), 28 saponins were quickly identified, most of which belonged to the oleanolic acid type. In the positive and negative ion mode, the additive ions of saponins were mainly [M+Na]^+^, [M+H]^+^, [M-H]^−^ and [M+HCOO]^−^. The mother nucleoside fragments were obtained by breaking or continuously breaking *O*-glycosyl or sugar groups, including glucose (162 Da), rhamnoside(146 Da), glucuronic acid (176 Da), galactose (162 Da), xylose (132 Da), and arabinose (132 Da) and so on. (Xia et al., 2019). In addition, the further characteristic fragment ions such as 191 Da and 174 Da were obtained by RDA rearrangement. For example, peak 43 showed a molecular formula of C_52_H_82_O_21_ (m/z 1087.5569 [M+HCOO]^−^. The fragment ion m/z 571.3712 [M-Rha-Glc-Glc-H]^−^ indicated that its mother nucleus was 30-noroleanolic acid, when combined with the loss of one rhamnose fragment ion to obtain m/z 941.4941 [M-Rha+HCOO]^−^ and one glucose fragment ion to get m/z 779.4745 [M-Rha-Glc+HCOO]^−^, which was preliminarily identified as ciwujianoside C1 (Figure 5D). Due to the existence of carboxyl structure, it was easy to lose fragment CO_2_ (44 Da) and gain m/z 391.1456 [M-Rha-Glc-Glc-Ara-CO_2_-H]^−^. A similar process also happened to ciwujianoside K (Figure 5E). Furthermore, the RDA rearrangement and partial loss of sugar phenomenon (Figures 5D,E) often occurred due to the presence of cycloolefin structure in the parent nucleus, which was a common and typical feature of saponins. The identification data of other compounds are shown in detail in Table 1.

### 3.4 Validation of the Quantitative Analytical Method

In the process of quantitative analysis, the contents of phenols and saponins from 29 different places were evaluated by the content determination of 10 reference compounds, which were selected because of their properties, structure, and content. The linearity, quantitative limit (LOQ), detection limit (LOD), repeatability, precision, stability, and recovery of the UPLC-QTRAP-MS/MS quantitative analysis method were verified. The integral peak area (Y) and concentration (X) of 10 reference compounds in six different concentration standard solutions were analyzed by linear regression analysis. The regression equation, determination coefficient, and linear range of the reference compounds were listed in Table 3. The LOD and LOQ under the present chromatographic conditions were determined at a signal-to-noise ratio (S/N) of about 3 and 10, respectively.

**TABLE 3:** The regression equation, linear range, limits of detection, and limits of quantification of 10 reference compounds.

No.	Compounds	Regression Equations	*R* ^2^	Linear ranges (μg/ml)	LOD (μg/ml)	LOQ (μg/ml)
A	Protocatechuic acid	*y* = 189.394x + 231726	0.9995	0.62–9.92	0.12	0.39
B	Chlorogenic acid	*y* = 364.782x − 52129	0.9990	0.72–22.88	0.27	0.89
C	Methyl 5-*O*-feruloylquinate	*y* = 0.2943x + 162.22	0.9997	0.75–24.0	0.21	0.71
D	Hyperoside	*y* = 147.357x + 108536	0.9992	0.97–30.72	0.48	1.61
E	Rutin	*y* = 92.062x + 22102	0.9998	0.68–21.76	0.29	0.97
G	3-*O*-*α*-L-Rhamnopyranosyl-(1→2)-*α*-L-arabinopyranoside-29-hydroxy oleanolic acid	*y* = 2.1906x + 4,328.5	0.9994	0.95–30.4	0.79	2.64
H	3-*O*-*β*-D-Glucopyranosyl-(1→2)-α-*L*-arabinopyranoside-29-hydroxy oleanolic acid	*y* = 7.9494x + 11670	0.9991	0.76–24.8	0.33	1.12
I	Ciwujianoside C4	*y* = 19.799x + 2,157.3	0.9995	1.1–35.2	0.28	0.92
J	Saponin P_E_	*y* = 1.4033x + 2,833.1	0.9994	1.0–32.0	0.66	2.17
K	Ciwujianoside K	*y* = 26.924x + 2,298.1	0.9996	1.2–38.4	0.82	2.73

Intra-day and inter-day changes were selected to evaluate the precision of the test. For the intra-day difference test, the mixed standard solution was analyzed within 1 day, while for the inter-day difference test, the solution was detected repeatedly in a continuous 3-day cycle. Variations were expressed by the relative standard deviation (RSD). Verification studies showed that the overall intra-day and inter-day variations (RSD) were less than 2.27% and 2.73%, respectively. In the stability test, the contents of 10 components in the sample solution were determined at 0, 2, 4, 8, 12, 24, and 48 h, respectively, and the RSD values were all less than 3.75%. In the repetitive test, the same samples were extracted six times and analyzed as mentioned above. The RSD values of 10 compounds were all less than 2.85%. The accuracy of the method was evaluated by recovery rate. A known amount of reference compounds was added to a certain amount of sample. The mixed solution of the standard was extracted and analyzed by the above-mentioned method. The experiment was repeated three times, and the accuracy of the method was good. The total recovery rate was 95.92%–101.04%, and the RSD was 1.64%–3.72% (Table 4). The results indicated that the determination of phenols and saponins by UPLC-QTRAP-MS/MS had high precision, accuracy, and sensitivity.

**TABLE 4:** The recovery of the 10 reference compounds.

No.	Compounds	Original (ng)	Spiked (ng)	Found (ng)	Recovery (%)	RSD (%, *n* = 3)
A	Protocatechuic acid	233.69	256.00	491.83	100.43	2.15
B	Chlorogenic acid	581.95	534.00	1,111.82	99.63	1.64
C	Methyl 5-*O*-feruloylquinate	420.68	450.00	853.53	98.03	2.38
D	Hyperoside	1,206.00	1,225.55	2,332.34	95.92	2.47
E	Rutin	675.00	680.00	1,371.26	101.20	1.95
G	3-*O*-*α*-L-Rhamnopyranosyl-(1→2)-*α*-*L*-arabinopyranoside-29-hydroxy oleanolic acid	1,769.15	1,710.00	3,352.50	96.36	2.57
H	3-*O*-*β*-D-Glucopyranosyl-(1→2)-*α*-*L*-arabinopyranoside-29-hydroxy oleanolic acid	841.66	852.50	1,685.86	99.51	2.42
I	Ciwujianoside C4	694.85	715.00	1,424.51	101.04	3.72
J	Saponin P_E_	1,285.40	1,250.00	2,468.97	97.38	2.84
K	Ciwujianoside K	1,670.57	1,650.00	3,330.87	100.31	1.87

### 3.5 Constituents Analysis of Samples

**FIGURE 6:**
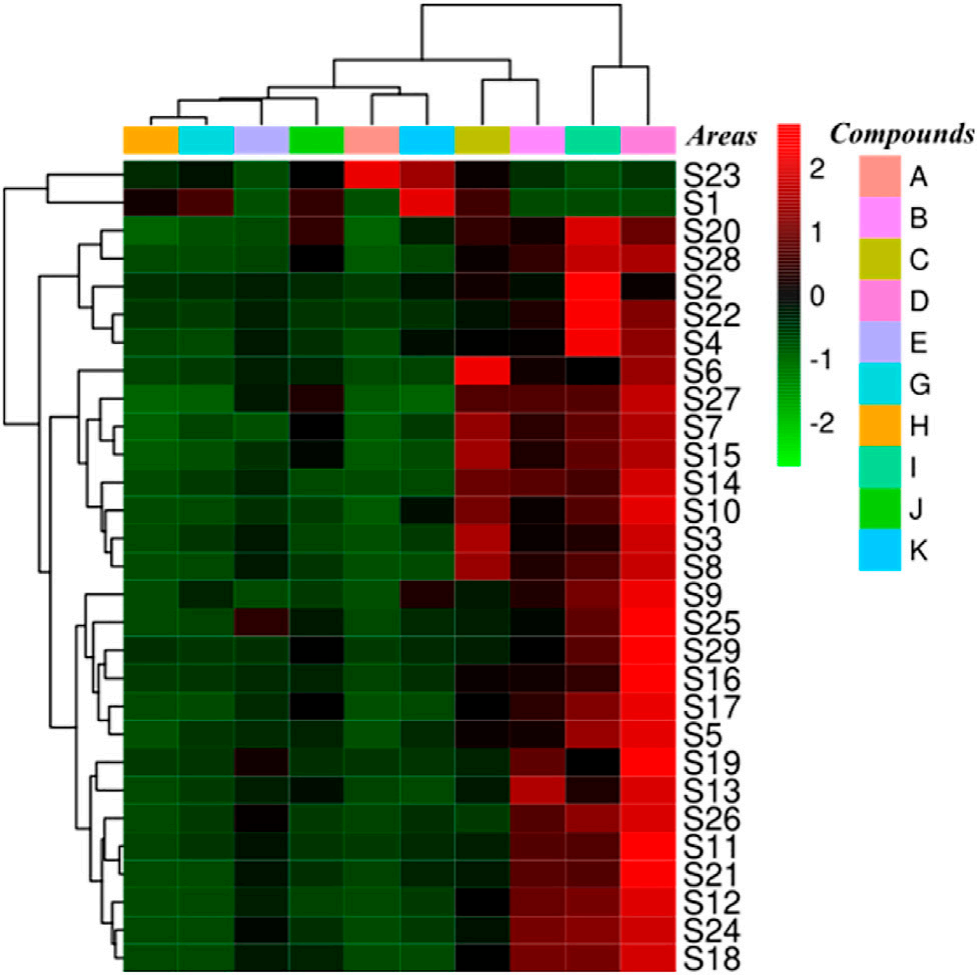
Cluster analysis of ESL from 29 different areas based on the content differences of 10 reference compounds.

Ten standard compounds in ESL samples from 29 locations were quantitatively determined by UPLC-QTRAP-MS/MS to comprehensively evaluate the contents of phenols and saponins. Each sample was analyzed three times to determine the mean contents (Table 5). The results showed that the contents of these compounds varied greatly among the samples collected from different habitats. The contents of protocatechuic acid (**A**, 8.41 ± 0.062 mg/g) and chlorogenic acid (**B**, 19.33 ± 0.392 mg/g) were the highest in S23 and S12, respectively, and methyl 5-*O*-feruloylquinate (**C**, 15.41 ± 0.173 mg/g), 3-*O*-*α*-L-rhamnopyranosyl-(1→2)-*α*-L-arabinopyranoside-29-hydroxy oleanolic acid (**G**, 16.20 ± 0.131 mg/g), 3-*O*-*β*-D-glucopyranosyl-(1→2)-*α*-L-arabinopyranoside-29-hydroxy oleanolic acid (**H**, 11.86 ± 0.324 mg/g), saponin P_E_ (**J**, 14.71 ± 0.094 mg/g), and ciwujianoside K (**K**, 30.60 ± 0.147 mg/g) were the highest in S1. The content of ciwujianoside C4 (**I**, 36.73 ± 0.582 mg/g) in S2, hyperoside (**D**, 36.56 ± 0.467 mg/g) and rutin (**E**, 11.11 ± 0.214 mg/g) in S19 was highest, respectively. Comprehensive analysis showed that the content of phenols in S19 was up to 69.89 ± 1.098 mg/g; the highest content of saponins in S1 was 74.28 ± 0.703 mg/g. This indicated that S19 and S1 will be better choices when these ingredients are required for further research. Cluster analysis (Figure 6) divided 29 locations into five categories. S2, S4, S20, S22, and S28 were the same categories. S1 and S23; S3, S6, S7, S8, S10, S14, S15, and S27; S5, S9, S16, S17, S25, and S29 were one category, respectively, and the rest were one category. These results suggested that different areas have different contents of phenols and saponins.

**TABLE 5:** The amounts of 10 reference compounds in ESL from different sources.

No.	Sources	Content (mg/g)									
		A	B	C	D	E	G	H	I	J	K
S1	Xiaoxing’anling	0.43 ± 0.007	0.71 ± 0.012	15.41 ± 0.173	1.47 ± 0.045	0.25 ± 0.006	16.20 ± 0.131	11.86 ± 0.324	0.91 ± 0.037	14.71 ± 0.094	30.60 ± 0.147
S2	Wangqing	0.39 ± 0.001	4.94 ± 0.071	10.90 ± 0.225	9.74 ± 0.044	2.78 ± 0.030	1.76 ± 0.020	1.11 ± 0.033	36.73 ± 0.582	1.74 ± 0.005	4.22 ± 0.056
S3	Huadian	0.39 ± 0.003	6.46 ± 0.253	14.00 ± 0.143	15.74 ± 0.253	3.53 ± 0.054	1.81 ± 0.032	1.07 ± 0.047	7.50 ± 0.018	1.33 ± 0.010	1.74 ± 0.038
S4	Huinan	0.61 ± 0.007	6.98 ± 0.036	5.76 ± 0.062	14.55 ± 0.117	3.79 ± 0.022	0.78 ± 0.002	1.22 ± 0.012	21.38 ± 0.360	2.32 ± 0.088	4.37 ± 0.109
S5	Huichun	0.76 ± 0.020	9.17 ± 0.192	8.40 ± 0.034	22.05 ± 0.308	3.53 ± 0.077	2.42 ± 0.055	0.48 ± 0.002	16.89 ± 0.205	3.68 ± 0.063	3.35 ± 0.088
S6	Harbin	0.63 ± 0.001	5.91 ± 0.050	15.39 ± 0.211	11.31 ± 0.135	2.44 ± 0.009	1.04 ± 0.023	0.65 ± 0.004	4.20 ± 0.037	2.25 ± 0.124	0.80 ± 0.026
S7	Linjiang	0.53 ± 0.015	6.11 ± 0.202	9.56 ± 0.042	10.66 ± 0.205	0.87 ± 0.002	1.46 ± 0.056	0.46 ± 0.013	7.84 ± 0.119	4.34 ± 0.099	1.60 ± 0.032
S8	Erdaobaihe	0.33 ± 0.001	5.25 ± 0.137	8.82 ± 0.020	10.54 ± 0.074	2.25 ± 0.028	0.60 ± 0.024	0.51 ± 0.010	6.39 ± 0.143	1.33 ± 0.026	0.42 ± 0.017
S9	Fenglin	1.19 ± 0.016	6.46 ± 0.106	3.30 ± 0.041	13.72 ± 0.012	1.64 ± 0.042	2.90 ± 0.077	1.33 ± 0.009	9.20 ± 0.311	2.09 ± 0.055	6.32 ± 0.097
S10	Baoqing	0.88 ± 0.005	6.32 ± 0.083	10.51 ± 0.067	15.22 ± 0.063	2.59 ± 0.111	1.75 ± 0.031	1.45 ± 0.033	9.04 ± 0.205	2.06 ± 0.077	4.19 ± 0.051
S11	Dunhua	0.73 ± 0.004	11.06 ± 0.108	2.55 ± 0.012	21.98 ± 0.197	3.44 ± 0.007	1.04 ± 0.013	0.41 ± 0.006	10.65 ± 0.248	1.17 ± 0.024	2.06 ± 0.018
S12	Anguo	0.95 ± 0.011	19.33 ± 0.392	9.97 ± 0.034	30.49 ± 0.439	4.94 ± 0.084	0.89 ± 0.037	0.56 ± 0.001	20.70 ± 0.414	1.66 ± 0.032	2.22 ± 0.037
S13	Shenyang	0.85 ± 0.006	13.74 ± 0.123	3.05 ± 0.015	15.53 ± 0.072	2.77 ± 0.021	1.26 ± 0.063	0.58 ± 0.013	6.83 ± 0.189	3.70 ± 0.113	0.55 ± 0.004
S14	Jingyu	0.60 ± 0.003	10.14 ± 0.272	10.69 ± 0.076	16.46 ± 0.098	2.67 ± 0.047	1.56 ± 0.019	0.46 ± 0.008	9.33 ± 0.083	0.89 ± 0.008	0.88 ± 0.003
S15	Antu	0.56 ± 0.014	6.89 ± 0.013	11.92 ± 0.162	12.92 ± 0.055	2.29 ± 0.016	0.68 ± 0.022	0.50 ± 0.016	9.40 ± 0.232	4.25 ± 0.022	1.08 ± 0.016
S16	Jiaohe	0.53 ± 0.010	6.87 ± 0.045	6.58 ± 0.043	21.28 ± 0.443	2.17 ± 0.031	1.28 ± 0.046	1.19 ± 0.003	8.51 ± 0.242	2.49 ± 0.008	1.68 ± 0.073
S17	Yanbian	0.74 ± 0.007	10.53 ± 0.101	7.60 ± 0.087	23.16 ± 0.615	3.36 ± 0.050	0.96 ± 0.039	1.52 ± 0.070	15.79 ± 0.477	6.96 ± 0.103	1.42 ± 0.066
S18	Ningan	0.74 ± 0.025	12.42 ± 0.135	5.36 ± 0.094	17.28 ± 0.250	3.59 ± 0.113	0.96 ± 0.053	0.92 ± 0.034	12.60 ± 0.233	2.80 ± 0.011	0.49 ± 0.035
S19	Huanren	0.85 ± 0.037	18.49 ± 0.374	2.89 ± 0.006	36.56 ± 0.467	11.11 ± 0.214	0.79 ± 0.022	0.31 ± 0.002	8.91 ± 0.201	1.33 ± 0.057	0.88 ± 0.072
S20	Hulin	0.54 ± 0.024	7.42 ± 0.044	9.12 ± 0.055	11.25 ± 0.015	1.92 ± 0.061	1.38 ± 0.056	0.48 ± 0.014	16.85 ± 0.003	9.09 ± 0.225	4.03 ± 0.106
S21	Chibei	0.80 ± 0.013	11.56 ± 0.067	3.20 ± 0.004	21.82 ± 0.327	3.73 ± 0.133	0.46 ± 0.018	0.52 ± 0.006	11.14 ± 0.381	1.95 ± 0.014	2.48 ± 0.054
S22	Tieli	0.70 ± 0.014	9.97 ± 0.110	4.35 ± 0.013	17.05 ± 0.088	3.27 ± 0.062	0.83 ± 0.030	1.22 ± 0.023	26.79 ± 0.694	1.60 ± 0.058	2.06 ± 0.003
S23	Fusong	8.41 ± 0.062	0.78 ± 0.005	2.89 ± 0.045	0.63 ± 0.004	0.10 ± 0.002	1.60 ± 0.043	0.93 ± 0.048	0.27 ± 0.002	2.22 ± 0.079	6.42 ± 0.039
S24	Dongning	0.88 ± 0.007	16.57 ± 0.201	5.38 ± 0.077	22.33 ± 0.171	5.99 ± 0.035	1.29 ± 0.085	1.26 ± 0.049	17.14 ± 0.401	2.92 ± 0.061	2.33 ± 0.044
S25	Raohe	1.11 ± 0.023	4.68 ± 0.093	3.18 ± 0.009	20.40 ± 0.389	8.48 ± 0.283	1.44 ± 0.069	1.00 ± 0.040	11.24 ± 0.239	3.80 ± 0.104	2.58 ± 0.112
S26	Tonghua	1.19 ± 0.034	11.70 ± 0.084	1.37 ± 0.003	20.15 ± 0.143	7.02 ± 0.075	1.28 ± 0.034	0.57 ± 0.012	15.37 ± 0.143	1.56 ± 0.040	2.04 ± 0.047
S27	Yanji	0.93 ± 0.010	7.99 ± 0.222	8.09 ± 0.041	12.47 ± 0.066	3.57 ± 0.016	0.67 ± 0.008	0.49 ± 0.005	8.38 ± 0.093	6.50 ± 0.137	0.49 ± 0.001
S28	Shihezi	0.57 ± 0.001	7.44 ± 0.099	5.87 ± 0.010	12.33 ± 0.132	1.43 ± 0.009	0.98 ± 0.011	1.13 ± 0.017	13.56 ± 0.670	5.41 ± 0.072	1.37 ± 0.046
S29	Bozhou	1.77 ± 0.043	5.67 ± 0.087	3.08 ± 0.075	18.76 ± 0.439	2.24 ± 0.017	2.07 ± 0.038	2.23 ± 0.035	9.59 ± 0.377	4.95 ± 0.091	2.49 ± 0.086

### 3.6 *α*-Glucosidase Inhibition Assay of ESL

*α*-Glucosidase inhibition assay was used to evaluate the hypoglycemic property of the extracted parts of ESL, respectively. As shown in Table 6, all extracts showed effective hypoglycemic activity. The results indicated that the hypoglycemic activity of different extracts was different. Among them, phenolic fraction had the best hypoglycemic activity (471.4 ± 17.7 μg/ml), followed by n-butanol (1004.3 ± 30.8 μg/ml), saponins (1094.0 ± 28.4 μg/ml), and alcohol extract (1386.4 ± 44.5 μg/ml). These results might be correlated with phenols content, suggesting that ESL could be a new plant source of natural hypoglycemic.

**TABLE 6:** Results of *α*-glucosidase inhibition assay.

	IC50 (μg/ml)	Inhibition (%) at 500 μg/ml
Phenolic fraction	471.4 ± 17.7	53.7 ± 7.0
Saponin fraction	1094.0 ± 28.4	22.7 ± 3.4
n-BuOH fraction	1004.3 ± 30.8	27.3 ± 2.6
Alcohol extract	1386.4 ± 44.5	18.4 ± 2.1

Values represent the mean ± SEM (*n* = 3).

## 4 Conclusion

This study established a new rapid and sensitive UPLC-QTOF-MS/MS method to identify phenols and saponins in ESL. Under the optimized conditions, 30 phenols and 28 saponins were detected and identified within 23.0 min *via* comparing the characteristic fragments of mass spectrometry with the information of the published literature (Figure 2; Table 1). Most phenolic acids were formed by dehydration condensation of one or two caffeic acids and one quinic acid or their derivatives. Due to the dissociation of oxygen methyl, carboxyl, and ester bond in their structures, losing OCH_3_/CH_3_ (31/15 Da), CO_2_ (44 Da), quinic acid (156 Da), caffeoyl (162 Da), caffeoyl (176 Da), or coumaroyl (146 Da) was their main cleavage characteristics. Flavonoids and saponins tended to be *O*-glycosides, and the most typical fragmentation behavior was the cleavage of the C-*O* bond. Their mother nucleus was obtained by destroying or continuously destroying *O*-glycosyl or sugar groups. The glycosyl of flavonoids mainly included rutinose (308 Da), galactose (162 Da), glucose (162 Da), and rhamnoside (146 Da), and saponins mainly lost glucose, rhamnoside, glucuronic acid (176 Da), galactose, xylose (132 Da), arabinose (132 Da), and so on. Because of the reverse Diels–Alder reaction (RDA), flavonoids were apt to produce characteristic fragments (CO, 28 Da), and saponins obtained characteristic ions such as 191 Da and 174 Da. Moreover, partial sugar loss was also a typical common feature of flavonoids and saponins. The exact or complete chemical structures of 30 compounds from the phenolic and saponin fractions of ESL were further clarified by nuclear magnetic resonance spectroscopy, of which 12 (including eight phenols) were isolated from this genus for the first time (Figure 4). To quantitatively determine 10 components in ESL from 29 different areas to evaluate the contents of phenols and saponins, a UPLC-QTRAP-MS/MS method was established. The results showed that the highest contents of phenols and saponins in S19 and S1 were 69.89 ± 1.098 and 74.28 ± 0.733 mg/g, respectively (Table 5). Cluster analysis (Figure 6) divided 29 locations into five categories, suggesting that different areas have different contents of phenols and saponins. The methodological investigation suggested that the established qualitative and quantitative methods could be used to evaluate the quality of ESL. In addition, the *α*-glucosidase inhibitory activity of phenolic fraction was the highest *in vitro* (Table 6), indicating that the phenolic content may be related to the hypoglycemic activity. It was suggested that ESL could be developed as a natural potential effective drug or functional food. However, its pharmacological effects *in vivo* and related mechanisms need to be further studied.

## Data Availability

The original contributions presented in the study are included in the article/Supplementary Material. Further inquiries can be directed to the corresponding author.
